# Development of evidence-based nursing recommendations for hand-foot syndrome in breast cancer chemotherapy patients

**DOI:** 10.3389/fonc.2026.1961472

**Published:** 2026-10-29

**Authors:** Liuqin Huang, Haixia Lu, Ying Zhou, Jiaqi Yuan, Dongmei Lan, Yan Xu

**Affiliations:** Liuzhou Hospital of Guangzhou Women and Children’s Medical Center, Liuzhou, Guangxi, China

**Keywords:** breast cancer, Delphi method, evidence-based nursing, hand-foot syndrome, nursing recommendations

## Abstract

**Objective:**

To develop evidence-based nursing recommendations for managing hand-foot syndrome (HFS) in breast cancer patients undergoing chemotherapy.

**Methods:**

Based on the JBI evidence-based healthcare model, relevant literature was systematically reviewed. Following group analysis and discussion, preliminary nursing recommendations were drafted. Subsequently, two rounds of Delphi expert consultation were conducted two weeks apart with twenty experts from breast surgery, dermatology, and pharmacy departments across ten tertiary Grade A hospitals located in Beijing, Shanghai, Guangzhou, Henan, Qinghai, and other regions. Expert feedback was used to revise and refine the recommendations, resulting in a final version.

**Results:**

The response rate for both rounds of expert consultation was 100%. The expert authority coefficients were 0.854 and 0.862, respectively, indicating strong expertise and reliable judgments. The coefficients of variation for expert ratings across the indicators ranged from 0.00 to 0.41 in the first round and from 0.00 to 0.13 in the second round. Kendall’s coefficients of concordance among experts across all indicators were 0.214 and 0.312, respectively (P<0.001), indicating a satisfactory level of expert consensus. The finalized nursing recommendations comprise 6 first-level domains (Assessment, Basic Prevention, Care During Treatment, Health Education, Psychosocial Support, and Follow-up) and 29 second-level items.

**Conclusion:**

The evidence-based nursing recommendations developed in this study demonstrate strong scientific rigor and practical applicability. It can serve as a reference for managing HFS in breast cancer patients undergoing chemotherapy and have potential for clinical implementation and broader dissemination.

## 1 Introduction

Hand-foot syndrome (HFS), also known as palmar-plantar erythrodysesthesia, is a common cutaneous adverse reaction affecting the hands and feet of breast cancer patients undergoing chemotherapy. Its typical symptoms include erythema, edema, numbness, tingling, blistering, or even ulceration of palms and soles, with severe cases potentially leading to nail loss and impairment of patients’ daily activities (1). The visible skin changes and associated physical discomfort can also contribute to psychological issues such as anxiety, depression, and reduced self-esteem, thereby diminishing patients’ quality of life. In severe cases, HFS may necessitate dose reduction, treatment interruption, or discontinuation of chemotherapy, ultimately impacting the treatment efficacy and prognosis (1, 2).

With the increasing incidence of breast cancer and widespread use of chemotherapeutic agents, the reported incidence of HFS ranges from 40% to 70% (1), posing a significant challenge in clinical nursing care. Although progress has been made in HFS-related nursing research, unified and systematic nursing recommendations are still lacking, leading to inconsistent clinical practices and variable outcomes (3). Therefore, developing scientific, standardized, and systematic nursing recommendations for managing HFS in breast cancer patients undergoing chemotherapy is crucial for improving patients’ prognosis and quality of life. This study aims to develop evidence-based nursing recommendations for managing HFS specifically in breast cancer patients undergoing chemotherapy through literature review and Delphi expert consultation method, thereby providing a scientific foundation for clinical nursing practice and contributing new insights and methodologies to evidence-based nursing.

## 2 Methods

### 2.1 Development of the preliminary nursing recommendations draft

#### 2.1.1 Establishment of the research team

The research team comprised nine members: one PhD candidate from the Southern Hospital JBI Evidence-Based Nursing Collaboration Centre who served as the project supervisor and provided overall guidance; two master students who had received systematic training in evidence-based practice, including the principal investigator, responsible for evidence collection and appraisal, expert liaison, and data analysis; and three associate chief nurses along with three senior nurses having extensive clinical and research experience, who were responsible for expert panel organization, quality control, coordination, and questionnaire development and distribution.

#### 2.1.2 Literature search

A comprehensive literature search was conducted across multiple databases and professional organization websites, including NGC, NICE, GIN, CMA Infobase, JBI, UpToDate, BMJ Best Practice, the Cochrane Library, RNAO, the Chinese Nursing Association, NCCN, the Chinese Anti-Cancer Association Committee of Oncology Nursing, PubMed, CINAHL, Web of Science, Embase, Wanfang Database, CNKI, VIP, and CBM. Following the “5S” evidence pyramid model (4), a systematic search was performed from database inception through April 2024. Chinese search terms included: breast cancer/breast malignancy; hand-foot syndrome/palmar-plantar erythrodysesthesia; management/intervention/treatment/nursing; and guidelines/expert consensus/clinical decision-making/best evidence/evidence summary/systematic review/meta-analysis/evidence-based medicine/evidence-based nursing. English search terms included: breast cancer/breast malignant*/breast tumors/breast neoplasm*; hand-foot syndrome/palmar-plantar erythrodysesthesia/skin reaction; management/intervention/treatment/nursing; and guidelines/expert consensus/clinical decision-making/best evidence/evidence summary/systematic review/meta-analysis/evidence-based medicine/evidence-based nursing. A combination of subject headings and free-text terms was used to comprehensively search all relevant databases. The search strategy was tailored to the specific features of each database to maximize retrieval. The complete search strategies for all databases and websites are provided in **Supplementary Table S1**. Inclusion criteria: ① Studies involving breast cancer patients undergoing chemotherapy; ② Publications related to HFS assessment, prevention, management, or follow-up, etc.; ③ Published clinical decision support documents, guidelines, expert consensuses, evidence summaries, systematic reviews, or high-quality randomized controlled trials (RCTs); ④ Publications in Chinese or English. Exclusion criteria: ① Duplicate publications; ② Conference abstracts and narrative reviews; ③ Publications with incomplete information or untraceable sources.

#### 2.1.3 Quality appraisal

The quality of included literature was independently appraised by two trained researchers, with disagreements resolved through consultation with a third methodological expert. The AGREE II instrument (5) was used to evaluate clinical guidelines, while JBI critical appraisal tools were applied to expert consensuses, systematic reviews, and RCTs. In case of conflicting evidence, priority was given to evidence with a higher level quality and more recent publication, with consideration given to domestic evidence.

The initial search yielded 31,631 records. After removing duplicates using NoteExpress, 6,443 records remained. Following title and abstract screening, 6,365 records were excluded, leaving 78 full-text articles for further assessment. These full-text articles were reviewed in detail, and 64 were excluded for reasons such as non-relevant study design, publication in languages other than Chinese or English, incomplete data, or conference abstracts. Consequently, 14 publications were included in the final analysis, comprising 1 clinical decision (6), 2 guidelines (7, 8), 4 expert consensuses (1, 9–11), 2 systematic reviews (12, 13), and 5 RCTs (14–18). These 14 publications formed the evidence base for the preliminary nursing recommendations draft, which were subsequently evaluated and refined through two rounds of Delphi expert consultation. The detailed quality appraisal results are provided in **Supplementary Table S2**.

#### 2.1.4 Formation of the preliminary draft

The research team extracted the best available evidence on HFS care from the 14 included documents, categorized and prioritized the evidence thematically, and supplemented it with practical clinical details to formulate the preliminary recommendations draft. The mapping of each recommendation to its supporting evidence and JBI evidence level is provided in **Supplementary Table S3**.

### 2.2 Optimization and refinement of the recommendations via Delphi expert consultation

#### 2.2.1 Development of the consultation questionnaire

The questionnaire comprised three parts: (1) instructions, including the purpose, significance, process, and completion guide); (2) a core section listing the 29 second-level items of the draft recommendations, with each item rated for importance and feasibility using a 5-point Likert scale (19), along with space for expert comments (additions, deletions, modifications); and (3) an expert information from collecting demographic data and self-rated familiarity with the topic.

#### 2.2.2 Expert selection

To ensure comprehensiveness, scientific validity, and practicality, experts in breast surgery/oncology nursing, dermatology, and pharmacy from tertiary Grade A hospitals across different regions (including Beijing, Shanghai, Guangdong, etc.) were invited to participate. Experts were purposively selected through the research team’s professional networks and recommendations from relevant academic societies. Inclusion criteria: ① associate senior professional title or above; ② bachelor’s degree or above; ③ more than 10 years of relevant frontline experience; and ④ voluntary participation. Ultimately, twenty experts from 10 tertiary Grade A hospitals in Beijing, Shanghai, Guangdong, Henan, Qinghai, Hunan, Guangxi, Hebei, Jiangsu, and Shenzhen were included.

#### 2.2.3 Conduct of expert consultation

Two rounds of expert consultation were conducted via WeChat (a social media platform) and email. Questionnaires were distributed, and reminders were sent after 10 working days. After the first round, items with an importance score ≤3.50 or a coefficient of variation ≥0.25 were discussed by the research team and considered for deletion or revision based on expert feedback. Feasibility scores and their CVs were also calculated as auxiliary references. A revised questionnaire was subsequently developed for the second round. In the second-round questionnaire, experts were provided with the aggregated results from the first round (mean importance and feasibility scores and CVs for each item) and a clear explanation of the modifications made by the research team. Experts were then asked to re-rate all items in light of this feedback. In each round, experts were asked to score each item for its importance and feasibility on a scale ranging from 1 to 5. Following the second round, analysis showed convergence of expert opinions, and the consultation process was concluded. The recommendations were then finalized based on expert input.

#### 2.2.4 Statistical analysis

Data were analyzed using Excel and SPSS version 22.0. The following metrics were calculated: expert response rate (positive coefficient) (20); expert authority coefficient (Cr), derived from judgment basis (Ca) and familiarity (Cs) using the formula Cr = (Ca+Cs)/2 with Cr≥0.70 indicating high authority; and the spread and agreement of expert opinions assessed using the coefficient of variation (CV) and Kendall’s coefficient of concordance (W) (21).

## 3 Results

### 3.1 Expert demographics

Twenty experts participated, including 5 males and 15 females; 12 nurses, 4 breast oncologists, 2 pharmacists, and 2 dermatologists. Clinical experience ranged from 10 to 33 years, with a mean of 19.0 ± 6.63 years. Four experts held senior professional title, and 16 held associate senior professional title. Three experts held doctoral degrees, 5 held master’s degrees, and 12 held bachelor’s degrees.

### 3.2 Expert engagement and authority

The response rates for both consultation rounds were 100% (positive coefficient=100%). The judgment basis coefficients (Ca) for the two consultation rounds were 0.836 and 0.841, while familiarity coefficients (Cs) were 0.873 and 0.882. The resulting authority coefficients were 0.854 and 0.862, respectively (both >0.700), indicating high expert authority and reliable judgments.

### 3.3 Expert consensus level

The coefficients of variation for expert opinions on the 29 items in the preliminary recommendations draft ranged from 0.00 to 0.41 in the first round and improved on the items in the revised recommendations in the second round, ranging from 0.00 to 0.13, indicating good and less divergence for expert opinions on the revised recommendations. Kendall’s coefficient of concordance W values were 0.214 and 0.312 (P<0.001) on the initial and revised recommendations, indicating expert consensus.

### 3.4 Expert consultation outcomes

Across both consultation rounds, 11 experts provided a total of 14 modification suggestions. The research team adopted 12 suggestions, resulting in the deletion of four second-level items, modification of four items, and addition of one new item. Key revisions included the following: Round 1: Two items with high CV (0.32, 0.41) were deleted. Based on expert feedback, two skin care items were merged. An item on avoiding repetitive activities was removed due to insufficient clarity. In addition, based on expert recommendations and supporting literature (1, 22, 23), an item recommending the use of 10% urea cream was added. Round 2: An item on assessment criteria was modified to incorporate multiple international grading systems (NCI, NCIC, WHO) and was merged with another assessment-related item. An item on assessment frequency was revised to emphasize continuous and dynamic assessment based on symptom changes. After two consultation rounds, expert consensus was achieved, resulting in the finalized recommendations with 6 first-level items and 29 second-level items, as summarized in **Table 1**.

**Table 1:** Nursing recommendations for hand-foot syndrome in breast cancer patients undergoing chemotherapy.

Primary indicator	Secondary indicator	Importance (mean ± SD)		Feasibility (mean ± SD)	
		Score	CV	Score	CV
1. Assessment	1.1 Assess HFS risk based on chemotherapy agents before treatment initiation; inform patients of potential skin reactions and provide basic HFS management knowledge.	5.00 ± 0.00	0.00	4.90 ± 0.31	0.06
	1.2 Assess patient’s skin condition before treatment; refer patients with pre-existing skin issues to dermatology to mitigate risk factors impacting anti-tumor therapy.	4.95 ± 0.22	0.05	5.00 ± 0.00	0.00
	1.3 Identify predisposing factors (e.g., diabetes, hypertension, anemia, peripheral neuropathy, hyperkeratosis, local wounds). Flag patients with such factors for close monitoring.	4.55 ± 0.51	0.11	5.00 ± 0.00	0.00
	1.4 Grade HFS severity using internationally recognized grading criteria (e.g., NCI, NCIC, WHO),with NCI-CTCAE 5.0 recommended as the preferred system. Assessment includes skin color, integrity, abnormal sensations, and pain in hands/feet.	4.90 ± 0.31	0.06	4.95 ± 0.22	0.05
	1.5 Conduct continuous, dynamic assessment during chemotherapy. Document findings thoroughly and adjust assessment frequency based on symptom changes.	4.75 ± 0.44	0.09	5.00 ± 0.00	0.00
	1.6 Educate patients on using the HFS-14 scale to self-assess the impact of HFS on quality of life as a patient-reported outcome measure.	4.30 ± 0.47	0.11	5.00 ± 0.00	0.00
2. Basic Prevention	2.1 Apply moisturizers to hands and feet, especially during autumn/winter.	4.85 ± 0.37	0.08	5.00 ± 0.00	0.00
	2.2 Use mild, fragrance-free, alcohol-free, non-steroidal bath/skin care products. Avoid skin irritants, solvents, or disinfectants.	4.80 ± 0.41	0.09	4.85 ± 0.37	0.08
	2.3 Wear loose, soft cotton clothing and comfortable shoes/socks. Minimize skin coverage to reduce sweating.	4.75 ± 0.44	0.09	4.80 ± 0.41	0.09
	2.4 Minimize and avoid direct sunlight exposure.	4.80 ± 0.41	0.09	4.30 ± 0.57	0.13
	2.5 Avoid contact with extremely hot or cold objects. Avoid prolonged bathing or hot water soaks.	4.80 ± 0.41	0.09	4.80 ± 0.41	0.09
	2.6 Avoid using adhesive bandages or dressings.	4.70 ± 0.47	0.10	4.80 ± 0.41	0.09
	2.7 Throughout treatment, avoid excessive pressure/friction on hands/feet, unprotected prolonged walking, and strenuous exercise.	4.65 ± 0.49	0.11	4.70 ± 0.47	0.10
3. Care During Treatment	3.1 Implement graded care based on HFS severity: ① Grade 0 (no symptoms): Basic prevention measures; assess once daily. ② Grade 1: Add Low-Level Laser Therapy (LLLT) to Grade 0 care; assess at least twice daily. ③ Grade ≥2: Notify attending physician promptly for collaborative treatment planning; assess at least twice daily; consider dermatology consultation. ④ Grade ≥3 with no improvement after one week of management: Convene multidisciplinary team discussion; assess at least three times daily.	5.00 ± 0.00	0.00	4.60 ± 0.50	0.11
	3.2 For tolerable HFS, emphasize importance of maintaining dose; for intolerable HFS, consider dose reduction or interruption.	5.00 ± 0.00	0.00	4.85 ± 0.37	0.08
	3.3 Apply local cooling during chemotherapy: use frozen gloves/socks (stored at -22 °C for 24h) from 15 minutes pre-chemotherapy to 15 minutes post-infusion.	4.80 ± 0.41	0.09	4.60 ± 0.50	0.11
	3.4 Instruct on topical antiperspirant use: once daily in week 1 of chemotherapy, then three times weekly. Avoid washing hands/bathing for 5 minutes after application to ensure skin penetration.	4.80 ± 0.41	0.09	4.50 ± 0.51	0.11
	3.5 For mild HFS, LLLT/PBM may be considered as an adjunctive nursing intervention to prevent symptom worsening.	4.90 ± 0.31	0.06	4.75 ± 0.55	0.12
	3.6 Apply 10% urea cream to hands/feet, three times daily.	4.80 ± 0.41	0.09	4.80 ± 0.41	0.09
4. Health Education	4.1 Employ diverse educational methods: lectures, bedside teaching, demonstrations, pamphlets, expert talks, patient support groups.	5.00 ± 0.00	0.00	5.00 ± 0.00	0.00
	4.2 Before chemotherapy, physicians explain risks and obtain informed consent. Primary nurses provide oral education on side effects, dietary requirements, post-discharge precautions to patients/families.	4.80 ± 0.41	0.09	4.60 ± 0.50	0.11
	4.3 During first chemotherapy cycle, primary nurses explain HFS manifestations, grading, prevention, and precautions.	4.80 ± 0.41	0.09	4.50 ± 0.51	0.11
	4.4 During subsequent cycles, assess HFS occurrence and patient adherence to basic prevention, providing targeted reinforcement.	4.90 ± 0.31	0.06	4.30 ± 0.47	0.11
	4.5 At discharge, ensure patients can recognize early HFS symptoms and know how to report them accurately for early intervention.	5.00 ± 0.00	0.00	5.00 ± 0.00	0.00
5. Psychosocial Support	5.1 Provide positive psychological intervention to foster constructive illness perceptions.	4.90 ± 0.31	0.06	4.30 ± 0.47	0.11
	5.2 Assess patient’s social support network and mobilize family/social support to the fullest extent.	4.90 ± 0.31	0.06	4.75 ± 0.55	0.12
6. Follow-up	6.1 Utilize mobile health technologies for remote symptom assessment at home, ensuring follow-up adherence and enhancing treatment continuity/effectiveness.	4.75 ± 0.44	0.09	4.80 ± 0.41	0.09
	6.2 Conduct follow-up every two weeks during treatment, monitoring symptom changes, medication adherence, and adverse effects for timely regimen adjustment.	5.00 ± 0.00	0.00	5.00 ± 0.00	0.00
	6.3 Continue follow-up for at least six months post-treatment, with routine follow-ups at months 3 and 6.	4.90 ± 0.31	0.06	4.30 ± 0.47	0.11

## 4 Discussion

### 4.1 Scientific rigor and reliability of the evidence-based recommendations

This study strictly adhered to the JBI model, systematically retrieving and integrating high-quality evidence from guidelines, clinical decisions, systematic reviews, and RCTs, thereby establishing a solid evidence foundation. Two rounds of Delphi consultation were conducted to refine the recommendations, ensuring professional consensus and clinical relevance. The 20 participating experts represented diverse yet relevant disciplines and possessed substantial professional experience. The 100% response rate and high authority coefficients (0.854 and 0.862) reflected strong engagement and expertise among the experts (24). The increased Kendall’s W and narrowed CV ranges (0.00-0.13) in the second round further indicated improved consensus and internal consistency. Together, these methodological strengths support the scientific rigor and reliability of the recommendations.

### 4.2 Feasibility and safety of the recommendations

The recommendations were developed with clinical practicality and patient acceptability in mind. Its measures are detailed and contextualized across six clearly defined domains. Feasibility scores for all items exceeded 4.0, indicating expert confidence in their implementation in clinical practice. The recommendations are consistent with FAME principles (Feasibility, Appropriateness, Meaningfulness, Effectiveness) (25), taking into account resource allocation and the need for individualized patient care.

### 4.3 Comprehensiveness and specificity of the recommendations

HFS significantly impacts breast cancer patients’ quality of life and treatment continuity. These recommendations integrate evidence, expert consensus, and the research team’s clinical experience into a comprehensive, six-domain framework, enabling full-cycle management. They address pre-, intra-, and post-chemotherapy phases through 29 specific items, including risk screening, proactive interventions (e.g., cooling, antiperspirants, 10% urea cream, low-level laser therapy [LLLT]), and technology-facilitated post-discharge support to improve patients’ adherence to their care recommendations.

The depth of these recommendations lies in its staged, differentiated precision care approach: dynamic risk assessment, severity-based intervention escalation (e.g., adding 10% urea cream for Grade 2, LLLT or wound care for Grade 3), and avoidance of a one-size-fits-all strategy. Incorporating the research team’s clinical experience addresses knowledge gaps (e.g., first-cycle education, symptom recognition at discharge) and psychological distress related to appearance changes (e.g., positive cognitive training, family support) (26).

By integrating multiple sources, the recommendations provide a holistic yet personalized framework, likely reducing HFS incidence, slowing progression, enhancing self-management, and improving quality of life. They established a scientific, practical, and compassionate standard for HFS care.

In addition, compared with previous clinical practice guidelines, these recommendations have several important differences. First, it is nursing-focused and emphasizes nurse-led non-pharmacological interventions. Second, whereas clinical practice guidelines focus more on treating symptoms, these recommendations cover assessment, skin care, education, psychosocial support, and follow-up, thereby providing more comprehensive nursing care. Third, these recommendations incorporate new evidence, such as the use of LLLT/PBM, which was not included in the guidelines. However, because this evidence is derived from a small pilot randomized controlled trial, LLLT/PBM is positioned as an adjunctive nursing intervention for mild HFS rather than a standard treatment, and further high-quality trials are needed before it can be recommended as routine care. These differences reflect the nursing scope and the specific needs of breast cancer patients, complementing rather than duplicating existing medical guidelines.

### 4.4 Future direction

Although this study successfully developed scientific and systematic evidence-based nursing recommendations for hand-foot syndrome (HFS) in breast cancer patients undergoing chemotherapy, several avenues for future research and clinical application remain.

First, the recommendations have not yet been tested in real-world clinical settings; therefore, future studies should focus on pilot implementation and clinical validation to evaluate its feasibility, acceptability, and effectiveness in diverse hospital environments. Second, while the Delphi method ensured expert consensus, the recommendations’ content may benefit from further refinement through multicenter clinical trials that assess patient outcomes such as HFS incidence, severity reduction, quality of life, and treatment adherence. Third, the integration of digital health tools, such as mobile health applications or telemedicine platforms, could be explored to enhance patient self-management and real-time symptom monitoring, particularly in outpatient or home-care settings. Fourth, cultural adaptations and translations of the recommendations may be necessary for international dissemination and application. Additionally, formal patient interviews were not conducted, and patient input was limited to unsystematic communication during routine care, the involvement of patients and caregivers in the co-design and evaluation of the recommendations should be prioritized to ensure person-centered care and to address the psychological and social dimensions of HFS more comprehensively.

It is also important to acknowledge a limitation regarding the evolving evidence base. The literature search for these recommendations were completed in April 2024. Since that time, new high-level evidence has emerged regarding pharmacological prevention of HFS, particularly the phase III trial of methylcobalamin (27) and the D-TORCH trial of topical diclofenac (28). Pharmacological agents such as celecoxib, steroids, methylcobalamin, and topical diclofenac were not included in these nursing recommendations, because the present study was intentionally designed to focus on non-pharmacological nursing interventions. Decisions regarding pharmacological prevention and management remain the responsibility of the treating physician. Nevertheless, nurses play a critical role in multidisciplinary care and should be aware of emerging pharmacological options so that they can collaborate with physicians to ensure patients are informed of these choices. Future updates of these nursing recommendations should therefore be developed in collaboration with medical oncologists to incorporate emerging pharmacological evidence where appropriate.

These efforts will contribute to the continuous improvement and global applicability of HFS nursing management in oncology care.

## 5 Conclusion

This study developed scientific, systematic, and practical evidence-based nursing recommendations for HFS in breast cancer patients undergoing chemotherapy, utilizing the Delphi method to achieve expert consensus. The recommendations demonstrate potential clinical applicability and promise for wide implementation, providing clear, standardized guidance for nursing staff while serving as a methodological reference for future evidence-based nursing practice and research in related fields.

## Data Availability

The original contributions presented in the study are included in the article/**Supplementary Material**. Further inquiries can be directed to the corresponding author.
