# Supplementary material for: Development of evidence-based nursing recommendations for hand-foot syndrome in breast cancer chemotherapy patients
Source: Front Oncol. 2026 Oct 29;16:1961472. doi: 10.3389/fonc.2026.1961472 (PMC13760333; doi:10.3389/fonc.2026.1961472)
Supplement: Supplementary file 1 [file DataSheet1.pdf]

Supplementary Table S1. Search strategies for all databases and websites

Part A. Chinese databases

Database	Search strategy
CNKI	Chinese search terms: SU=(‘乳腺癌’+‘乳腺恶性肿瘤’+‘乳房癌’+‘乳癌’)AND SU=(‘手足综合征’+‘掌趾感觉丧失性红斑综合征’+‘掌趾感觉丧失性红斑’+‘皮肤反应’) AND SU=(‘管理’+‘干预’+‘治疗’+‘护理’)AND SU=(‘指南’+‘专家共识’+‘临床决策’+‘最佳证据’+‘证据总结’+‘证据汇总’+‘系统综述’+‘系统评价’+‘Meta 分析’+‘循证医学’+‘循证护理’+‘随机对照试验’+‘随机对照’+‘RCT’)<br>English translation of key terms: breast cancer/breast malignancy; hand-foot syndrome/palmar-plantar erythrodysesthesia/skin reaction; management/intervention/treatment/nursing; guidelines/expert consensus/clinical decision-making/best evidence/evidence summary/systematic review/meta-analysis/evidence-based medicine/evidence-based nursing/randomized controlled trial/RCT.
Wanfang	M=(乳腺癌+乳腺恶性肿瘤+乳房癌+乳癌) AND M=(手足综合征+掌趾感觉丧失性红斑综合征+掌趾感觉丧失性红斑+皮肤反应) AND M=(管理+干预+治疗+护理) AND M=(指南+专家共识+临床决策+最佳证据+证据总结+系统综述+系统评价+Meta 分析+循证医学+循证护理+随机对照试验+随机对照+RCT)<br>Search terms are the same as those used in CNKI; field operators were adapted to VIP syntax.
CBM	(乳腺癌 OR 乳腺恶性肿瘤 OR 乳房癌 OR 乳癌) AND (手足综合征 OR 掌趾感觉丧失性红斑综合征 OR 掌趾感觉丧失性红斑 OR 皮肤反应) AND (管理 OR 干预 OR 治疗 OR 护理) AND (指南 OR 专家共识 OR 临床决策 OR 最佳证据 OR 证据总结 OR 系统综述 OR 系统评价 OR Meta 分析 OR 循证医学 OR 循证护理 OR 随机对照试验 OR 随机对照 OR RCT)<br>Search terms are the same as those used in CNKI; field operators were adapted to CBM syntax.

Part B. English databases

Database	Search strategy
PubMed	#1 Breast Neoplasms[mesh]<br>#2 "breast neoplasm" OR "breast tumor" OR "breast cancer" OR "breast carcinoma" OR "breast tumour" OR "breast sarcoma" OR "breast malignan"<br>#3 breast dcis OR breast ductal OR breast infiltrating OR breast intraductal OR breast lobular OR breast medullary<br>#4 #1 OR #2 OR #3<br>#5 Mammary Neoplasms[mesh]<br>#6 "mammary neoplasm" OR "mammary cancer" OR "mammary carcinoma" OR "mammary tumour" OR "mammary tumor" OR "mammary adenocarcinoma" OR "mammary sarcoma" OR "mammary malignan"<br>#7 #5 OR #6<br>#8 #4 OR #7<br>#9 Hand-foot syndrome[mesh]<br>#10 "palmar-plantar erythrodysesthesia" OR "hand-foot syndrome" OR "skin reaction"<br>#11 #9 OR #10<br>#12 management[mesh]<br>#13 intervention OR treat OR nursing OR manage* OR care<br>#14 #12 OR #13<br>#15 guidelines OR expert consensus OR clinical decision-making OR best evidence OR evidence summary OR evidence aggregation OR systematic review OR systematic evaluation OR meta-analysis<br>#16 "evidence-based practice" OR "evidence-based nursing practice" OR "evidence-based health care" OR "evidence-based medicine" OR evidence-based medicine OR evidence-based nursing<br>#17 "randomized controlled trial" OR "randomised controlled trial" OR "RCT" OR randomized OR randomised<br>#18 #15 OR #16 OR #17<br>#19 #8 AND #11 AND #14 AND #18

CINAHL	(MH "Breast Neoplasms") OR (TI "breast cancer" OR AB "breast cancer") OR (TI "breast neoplasm" OR AB "breast neoplasm") AND (MH "Hand-Foot Syndrome") OR (TI "hand-foot syndrome" OR AB "hand-foot syndrome") OR (TI "palmar-plantar erythrodysesthesia" OR AB "palmar-plantar erythrodysesthesia") AND (MH "Management") OR (TI management OR AB management) OR (TI nursing OR AB nursing) AND (TI guideline* OR AB guideline*) OR (TI "expert consensus" OR AB "expert consensus") OR (TI "systematic review" OR AB "systematic review") OR (TI "meta-analysis" OR AB "meta-analysis") OR (TI "randomized controlled trial" OR AB "randomized controlled trial") OR (TI "randomised controlled trial" OR AB "randomised controlled trial")
Web of Science	TS=("breast cancer" OR "breast neoplasm" OR "breast tumor" OR "breast carcinoma") AND TS=("hand-foot syndrome" OR "palmar-plantar erythrodysesthesia" OR "skin reaction") AND TS=(management OR intervention OR nursing OR treatment OR care) AND TS=(guideline OR consensus OR "clinical decision" OR "best evidence" OR "evidence summary" OR "systematic review" OR "meta-analysis" OR "evidence-based" OR "randomized controlled trial" OR "randomised controlled trial" OR RCT)
Embase	#1 'breast tumor'/exp<br>#2 'breast cancer':ti,ab,kw OR 'breast neoplasm':ti,ab,kw OR 'breast carcinoma':ti,ab,kw<br>#3 #1 OR #2<br>#4 'hand foot syndrome'/exp<br>#5 'palmar plantar erythrodysesthesia':ti,ab,kw OR 'hand foot syndrome':ti,ab,kw OR 'skin reaction':ti,ab,kw<br>#6 #4 OR #5<br>#7 'management'/exp OR 'nursing'/exp<br>#8 management:ti,ab,kw OR intervention:ti,ab,kw OR nursing:ti,ab,kw OR treatment:ti,ab,kw<br>#9 #7 OR #8<br>#10 'practice guideline'/exp OR 'consensus'/exp OR 'systematic review'/exp OR 'meta analysis'/exp OR 'randomized controlled trial'/exp<br>#11 guideline*:ti,ab,kw OR consensus:ti,ab,kw OR 'systematic review':ti,ab,kw OR 'meta analysis':ti,ab,kw OR 'evidence based':ti,ab,kw OR 'randomized controlled trial':ti,ab,kw OR 'randomised controlled trial':ti,ab,kw<br>#12 #10 OR #11<br>#13 #3 AND #6 AND #9 AND #12
Cochrane Library	#1 MeSH descriptor: [Breast Neoplasms] explode all trees<br>#2 ("breast cancer" OR "breast neoplasm" OR "breast tumor"):ti,ab,kw<br>#3 #1 OR #2<br>#4 MeSH descriptor: [Hand-Foot Syndrome] explode all trees<br>#5 ("hand-foot syndrome" OR "palmar-plantar erythrodysesthesia" OR "skin reaction"):ti,ab,kw

	#6 #4 OR #5<br>#7 (management OR intervention OR nursing OR treatment OR care):ti,ab,kw<br>#8 (guideline* OR consensus OR "systematic review" OR "meta-analysis" OR "evidence-based" OR "randomized controlled trial" OR "randomised controlled trial"):ti,ab,kw<br>#9 #3 AND #6 AND #7 AND #8

Part C. Guideline websites

Website	Keywords
NGC	"hand-foot syndrome", "breast cancer", "chemotherapy", "nursing", "management", "randomized controlled trial"
NICE	"hand-foot syndrome", "breast cancer", "chemotherapy", "nursing", "management", "randomized controlled trial"
GIN	"hand-foot syndrome", "breast cancer", "chemotherapy", "nursing", "management", "randomized controlled trial"
CMA Infobase	"hand-foot syndrome", "breast cancer", "chemotherapy", "nursing", "management", "randomized controlled trial"
JBI	"hand-foot syndrome", "breast cancer", "chemotherapy", "nursing", "management", "randomized controlled trial"
UpToDate	"hand-foot syndrome", "breast cancer", "chemotherapy", "nursing", "management", "randomized controlled trial"
BMJ Best Practice	"hand-foot syndrome", "breast cancer", "chemotherapy", "nursing", "management", "randomized controlled trial"
RNAO	"hand-foot syndrome", "breast cancer", "chemotherapy", "nursing", "management", "randomized controlled trial"
Chinese Nursing Association	"手足综合征", "乳腺癌", "化疗", "护理", "管理", "随机对照试验"
NCCN	"hand-foot syndrome", "breast cancer", "chemotherapy", "nursing", "management", "randomized controlled trial"
Chinese Anti-Cancer Association Committee of Oncology Nursing	"手足综合征", "乳腺癌", "化疗", "护理", "管理", "随机对照试验"
