# Supplementary material for: Development of evidence-based nursing recommendations for hand-foot syndrome in breast cancer chemotherapy patients
Source: Front Oncol. 2026 Oct 29;16:1961472. doi: 10.3389/fonc.2026.1961472 (PMC13760333; doi:10.3389/fonc.2026.1961472)
Supplement: Supplementary file 2 [file DataSheet2.pdf]

Supplementary Table S2.Literature Quality Assessment Results

Table 1 Quality evaluation of included guidelines

Included guideline	Scope and purpose	Stakeholder involvement	Rigour of development	Clarity of presentation	Applicability	Editorial independence	Number of domains <30%	Number of domains > 60%
M.E. Lacouture et al.	100	77.8	87.5	97.22	81.3	100.0	0	6
Chinese Anti-Cancer Association Oncologic Nursing Committee	100	88.9	83.3	94.44	77.1	88.5	0	6

Table 2 Evaluation results of expert consensus

Included consensus	Was the source of the viewpoint clearly indicated?	Was the viewpoint from influential experts in the field?	Was the viewpoint centered on the interests of the relevant population?	Was the conclusion based on analysis? Was the expression logical?	Were other existing literature referenced?	Were there inconsistencies with previous literature?
Breast Cancer Group, Society of Oncologists, Chinese Medical Doctor Association	Yes	Yes	Yes	Yes	Yes	Yes
Jade Cury-Martins et al	Yes	Yes	Yes	Yes	Yes	Yes
Shanghai Medical Association Dermatology and Venereology Branch	Yes	Yes	Yes	Yes	Yes	Yes
Guangdong Nursing Association	No	Yes	Yes	Yes	Yes	Yes

Table 3 Evaluation results of systematic reviews

Systematic review	Was the evidenceb-ased question clear and explicit?	Were the inclusion criteria appropriat-e?	Was the search strategy appropriat-e?	Were the literature sources appropri-ate?	Was the quality assessment standard appropriate ?	Was the quality assessment completed independent-ly?	Measures to reduce data extraction errors	Was the method for combining studies appropriate?	Was publicati on bias-assessed ?	Were recommen dations based on results?	Were suggestions for future research appropriate?
Xuan-Zhang Huang et al.	Yes	Yes	Yes	Yes	Yes	Yes	Yes	Yes	Yes	Yes	Yes
Lígia Traldi Macedo et al.	Yes	Yes	Yes	Yes	Yes	Yes	Yes	Yes	Yes	Yes	Yes

Table 4 Evaluation results of randomized controlled trials

Item	Xie Tan et al.	N. Kearney et al.	S. Latifyan et al.	Zhang Qing et al.	Chu Yanxiang et al.
Was the randomization appropriate?	Yes	Yes	Yes	Yes	Yes
Was allocation concealment used?	Yes	No	No	Yes	Yes
Were baseline data comparable?	No	Yes	Yes	Yes	Yes
Were participants blinded?	Yes	No	Yes	Yes	Yes
Were intervention providers blinded?	No	No	Yes	Yes	Yes
Were outcome assessors blinded?	Yes	No	Yes	Yes	Yes
Were other interventions consistent between groups?	Yes	Yes	Yes	Yes	Yes
Was follow-up complete?	Yes	Yes	Yes	Yes	Yes
Were all participants included in the analysis?	Yes	Yes	Yes	Yes	Yes
Were outcome measures consistent?	Yes	Yes	Yes	Yes	Yes
Were outcome measurement methods reliable?	Yes	Yes	Yes	Yes	Yes
Was the data analysis method appropriate?	Yes	Yes	Yes	Yes	Yes
Was the trial design appropriate?	Yes	Yes	Yes	Yes	Yes
