# Supplementary material for: Development of evidence-based nursing recommendations for hand-foot syndrome in breast cancer chemotherapy patients
Source: Front Oncol. 2026 Oct 29;16:1961472. doi: 10.3389/fonc.2026.1961472 (PMC13760333; doi:10.3389/fonc.2026.1961472)
Supplement: Supplementary file 3 [file DataSheet3.pdf]

Supplementary Table S3. Mapping of recommendation items to supporting evidence

Note: recommendation items correspond to Table 1 in the main text. Evidence sources are numbered according to the reference list.Evidence levels were graded according to the JBI (Joanna Briggs Institute) Levels of Evidence (2014).

recommendation item	Recommendation	Source	JBI level
1.1	Assess HFS risk based on chemotherapy agents before treatment initiation	Ref 1, 7, 9,11	Level 5.b
1.2	Assess patient’s skin condition before treatment; refer to dermatology	Ref 1, 6, 7, 11	Level 5.b
1.3	Identify predisposing factors and flag for close monitoring	Ref 1, 7, 11	Level 5.b
1.4	Grade HFS severity using NCI-CTCAE 5.0; assess skin color, integrity, sensations, pain	Ref 1, 6, 9,11	Level 5.b
1.5	Conduct continuous, dynamic assessment during chemotherapy	Ref 1, 10,11,12	Level 1.a
1.6	Educate patients on using the HFS-14 scale to self-assess the impact of HFS on quality of life as a patient-reported outcome measure.	Ref 7,11	Level 5.b
2.1	Apply moisturizers to hands and feet	Ref 1, 7, 8, 9,10	Level 5.b
2.2	Use mild, fragrance-free, alcohol-free skin care products	Ref 1, 6,7, 8, 9, 10,11	Level 5.b
2.3	Wear loose, soft cotton clothing and comfortable shoes/socks	Ref 7, 8, 9, 10,11	Level 5.b
2.4	Minimize and avoid direct sunlight exposure	Ref 1, 8, 9,11	Level 5.b
2.5	Avoid extremely hot or cold objects; avoid prolonged hot water soaks	Ref 1, 8, 10,11	Level 5.b
2.6	Avoid using adhesive bandages or dressings	Ref 8, 9	Level 5.b
2.7	Avoid excessive pressure/friction and strenuous exercise	Ref 7, 8, 9, 10	Level 5.b
3.1	Implement graded care based on HFS severity (Grade 0–≥3)	Ref 1,7,10, 16, 18	Level 1.c
3.2	For tolerable HFS maintain dose; for intolerable HFS consider dose reduction	Ref 9, 10,12,13	Level 1.a

3.3	Apply local cooling during chemotherapy for taxane/liposomal doxorubicin regimens	Ref 1,7,9,10,11,14,16	Level 1.c
3.4	Instruct on topical antiperspirant use during chemotherapy	Ref 9, 10	Level 5.b
3.5	For mild HFS, provide LLLT or PBM	Ref 16	Level 1.c
3.6	Apply 10% urea cream to hands/feet three times daily	Ref 7, 8, 10, 11, 18	Level 1.c
4.1	Employ diverse educational methods	Ref 9, 17	Level 1.c
4.2	Physicians explain risks; nurses provide oral education	Ref 7, 9	Level 5.b
4.3	During first cycle, nurses explain HFS manifestations, grading, prevention	Ref 7, 9, 18	Level 1.c
4.4	During subsequent cycles, assess HFS occurrence and adherence	Ref 7,9, 18	Level 1.c
4.5	At discharge, ensure patients recognize early HFS symptoms	Ref 7, 9, 11	Level 5.b
5.1	Provide positive psychological intervention	Ref 9, 17	Level 1.c
5.2	Assess social support network and mobilize family support	Ref 9	Level 5.b
6.1	Utilize mobile health technologies for remote symptom assessment	Ref 15	Level 1.c
6.2	Conduct follow-up every two weeks during treatment	Ref 6, 18	Level 1.c
6.3	Continue follow-up for at least six months post-treatment	Ref 1, 11	Level 1.c
